# 

**Published:** 1898-08

**Authors:** 


### CONTENTS.

**ORIGINAL COMMUNICATIONS.** PAGE

Restoration with Porcelain in Fracture of the Teeth. By L. Foster Jack, M.D., D.D.S., Philadelphia . . . 493
Empyema of the Antrum. By Dr. Emma E. Musson . . . 496
Physical Conditions that determine Choice of Filling-Materials. By S. Blair Luckie, D.D.S., Chester, Pa. . . . 503
American Dentistry Abroad. By L. C. Bryan, D.D.S., F.B.D.C., etc., Basel and Davos, Switzerland . . . 509

**ABSTRACTS AND TRANSLATIONS.**

The Administration of Anæsthetics . . . 514

**REPORTS OF SOCIETY MEETINGS.**

Odontological Society of Pennsylvania . . . 519
The New York Institute of Stomatology . . . 528
Academy of Stomatology . . . 534

**EDITORIAL.**

Dr. Black's Review . . . 544
A Reply from the New Jersey Secretary . . . 547
A Move in the Wrong Direction . . . 549

**BIBLIOGRAPHY** . . . 550

**OBITUARY.**

Resolutions of Respect to Dr. Alonzo Boice . . . 557

**DOMESTIC CORRESPONDENCE.**

Reply to Editorial, May, 1898 . . . 558

**NOTES AND COMMENTS** . . . 561

**CURRENT NEWS.**

National Dental Association . . . 563
National Association of Dental Faculties . . . 563
Harvard Dental Alumni Association . . . 564

